# CE–MS for metabolomics: Developments and applications in the period 2014–2016

**DOI:** 10.1002/elps.201600370

**Published:** 2016-10-31

**Authors:** Rawi Ramautar, Govert W. Somsen, Gerhardus J. de Jong

**Affiliations:** ^1^ Division of Analytical Biosciences, LACDRLeiden UniversityLeidenThe Netherlands; ^2^ Division of BioAnalytical ChemistryVrije Universiteit AmsterdamAmsterdamThe Netherlands; ^3^ Biomolecular Analysis, Utrecht Institute for Pharmaceutical SciencesUtrecht UniversityUtrechtThe Netherlands

**Keywords:** Biomedical and clinical, Food, Metabolomics, Microbial and plant, Technological developments

## Abstract

CE–MS can be considered a useful analytical technique for the global profiling of (highly) polar and charged metabolites in various samples. Over the past few years, significant advancements have been made in CE–MS approaches for metabolomics studies. In this paper, which is a follow‐up of a previous review paper covering the years 2012–2014 (*Electrophoresis* 2015, 36, 212–224), recent CE–MS strategies developed for metabolomics covering the literature from July 2014 to June 2016 are outlined. Attention will be paid to new CE–MS approaches for the profiling of anionic metabolites and the potential of SPE coupled to CE–MS is also demonstrated. Representative examples illustrate the applicability of CE–MS in the fields of biomedical, clinical, microbial, plant, and food metabolomics. A complete overview of recent CE–MS‐based metabolomics studies is given in a table, which provides information on sample type and pretreatment, capillary coatings, and MS detection mode. Finally, general conclusions and perspectives are given.

**Abbreviations**
DFMO: difluoromethylornithine
HCC: hepatocellular carcinoma
HD: Huntington's disease
MSI: multisegment injection
PTH: poly‐(*N*,*N*,*N*’,*N*’‐tetraethyldiethylenetriamine, *N*‐(2‐hydroxypropyl) methacrylamide

## 1 Introduction

The ultimate aim of metabolomics research is to effectively address a specific biological or clinical problem 1. To achieve this goal, state‐of‐the‐art analytical separation techniques are increasingly used for the global profiling of (endogenous) metabolites in biological samples 2. At present, the profiling of endogenous metabolites is commonly performed with LC coupled to MS. Regardless of important developments in LC column technology and methodology, the selective and efficient analysis of (highly) polar ionogenic metabolites is still highly challenging.

In CE, compounds are separated on the basis of differences in their intrinsic electrophoretic mobility, which is dependent on the charge and size of the analyte. Therefore, CE is highly suited for the analysis of polar and charged metabolites. Moreover, as the separation mechanism of CE is fundamentally different from chromatographic‐based separation techniques, a complementary view on the composition of metabolites present in a given biological sample is provided 3, 4, 5. Andreas et al. used a combination of RP LC–MS, GC–MS, CE–MS, and ^1^H‐NMR spectroscopy for global metabolic profiling of human breast milk 5. The combined approach allowed the analysis of more than 700 metabolites in pretreated breast milk samples. The largest number of metabolites was detected by RP LC–MS, while CE–MS allowed the selective analysis of amino acids in breast milk. GC–MS showed to be very effective for the profiling of volatile compounds and short‐chain fatty acids and NMR provided highly repeatable metabolic profiles for compounds, such as monosaccharides, disaccharides, human milk oligosaccharides, amino acids, and some organic acids.

In comparison to other analytical techniques, the use of CE–MS in the field of metabolomics is still relatively low 2, 6. CE–MS is often considered a technically challenging approach and constraints such as poor concentration sensitivity, migration time variability, and method robustness may have hindered widespread use of this technology. However, it is of interest to note that CE–MS has been used for the global and reproducible profiling of native peptides and (endogenous) metabolites in a clinical setting for more than a decade now 7, 8, 9, 10. For example, Mischak and co‐workers have profiled native peptides in more than 20 000 human urine samples by CE–MS at different laboratories with an acceptable interlaboratory reproducibility 8, 11. The first CE–MS approaches used for global metabolic profiling of biological samples were introduced by Soga et al. 12, 13. On the basis of these developed CE–MS strategies, authors initiated Human Metabolome Technologies (HMT), a Japan‐based company which is providing methods and workflows for CE–MS‐based metabolomics. So far, the HMT approaches and protocols have been used for a wide range of metabolomics studies 9, 14, 15, 16. Overall, the studies outlined above clearly demonstrate the usefulness and added value of CE–MS in the field of metabolomics.

Over the past few years, CE–MS has gained increased attention for metabolomics studies, especially with the development of novel interface designs to improve the concentration sensitivity 17, 18, 19, 20. This paper, which is a follow‐up of our previous CE–MS‐based metabolomics reviews 21, 22, 23, 24, provides an overview of CE–MS approaches and strategies for metabolomics studies as reported over the past 2 years. Emerging technological developments used in CE–MS for metabolomics are discussed, that is, SPE coupled on‐line to CE–MS and special systems for the profiling of anionic metabolites. The recent CE–MS‐based metabolomics studies are summarized in a table and selected illustrative examples are discussed in detail. Finally, some general conclusions and perspectives are provided. Aspects related to data analysis and identification of metabolites are not covered in this paper. The reader is referred to more dedicated literature for an overview concerning these topics 25, 26, 27, 28.

## 2 Technological developments

The use of CE–MS for the analysis of anionic metabolites at high pH BGE conditions has proven to be challenging as compared to the analysis of cationic metabolites at low‐pH BGE conditions. The formation of corona discharge is often an issue when applying MS in negative‐ionization mode and in CE–MS using a classical sheath‐liquid interface corrosion may be expected from stainless steel ESI spray needles in reversed polarity mode at high pH separation conditions 29, 30. Soga and co‐workers demonstrated that the performance of CE–MS using a cationic coated capillary for anionic metabolic profiling was significantly improved by replacing stainless steel for platinum as ESI spray needle 30. However, a platinum sprayer is not needed for anionic metabolic profiling using normal CE polarity. Kok et al. has evaluated various BGE and sheath‐liquid compositions using normal polarity CE to improve the detection sensitivity for anionic metabolic profiling 4, 31. The use of triethylamine in the BGE and sheath‐liquid appeared to be an effective way to enhance metabolite responses in CE with MS detection in negative‐ionization mode.

**Figure 1:**
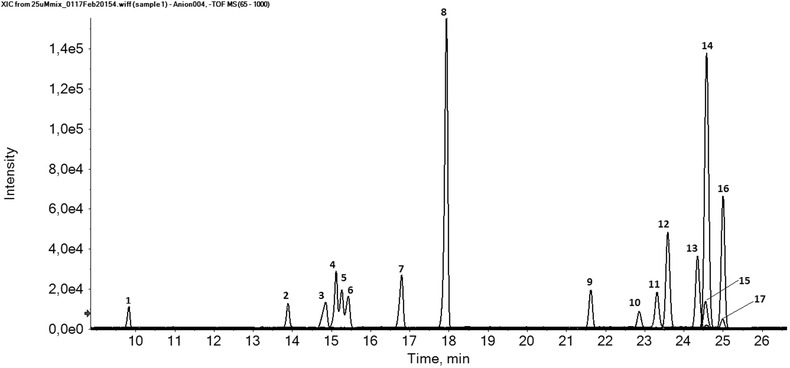
Performance of CE–MS using a sheathless porous tip sprayer for anionic metabolic profiling. Multiple extracted ion electropherograms for the metabolite test mixture (25 μM) obtained with sheathless CE–MS in negative‐ion mode using a porous tip sprayer. Peaks: 1, 2‐Naphtol‐3,6‐disulfonic acid; 2, d(+)2‐phosphoglyceric acid; 3, d‐ribose‐5‐phosphate; 4, d‐glucose‐1‐phosphate; 5, d‐glucose‐6‐phosphate; 6, d‐fructose‐6‐phosphate; 7, inosine 5’‐monophosphate (IMP); 8, guanosine 3’,5’‐cyclic monophosphate (cGMP); 9, guanosine 5’‐monophosphate; 10, citric acid; 11, trimesic acid; 12, isocitric acid; 13, gluconic acid; 14, adenosine 3’,5’‐cyclic monophosphate (cAMP); 15, 2‐hydroxybutyric acid; 16, b‐diphosphopyridine nucleotide (NAD+); 17, 3‐hydroxybutyric acid. Experimental conditions: BGE, 10% acetic acid (pH 2.2); separation voltage, −30 kV (+0.5 psi applied at the inlet of the CE capillary); sample injection, 2.0 psi for 60 s. Reproduced from 32 with permission.

In our previous reviews, we have highlighted the potential of CE–MS using a sheathless porous tip interface for highly sensitive profiling of cationic metabolites 23, 24. Recently, Gulersonmez et al. has evaluated this method for the profiling of anionic metabolites by using the same experimental conditions as for the profiling of cationic metabolites 32, that is a bare fused‐silica capillary, 10% acetic acid as BGE (pH 2.2), and nanospray ESI source. However, the electrophoretic separation voltage and MS detection polarity were reversed. A diverse range of anionic metabolite classes could be profiled under these conditions, including sugar phosphates, nucleotides, and organic acids (Fig. 1). An injection volume of circa 20 nL resulted in nanomolar detection limits, which correspond to a significant improvement as compared to the micromolar detection limits typically obtained with classical sheath‐liquid CE–MS methods. Structural isomers of phosphorylated sugars could be selectively analyzed without using any derivatization. For test compounds, RSDs for migration times and peak areas were below 2 and 11%, respectively. The method was used for profiling of anionic metabolites in extracts of cultured glioblastoma cells. It is important to note that a low‐pH BGE may not be the most optimal for global profiling of anionic metabolites as only those compounds can be analyzed that are (partially) negatively charged under the used separation conditions.

**Figure 2:**
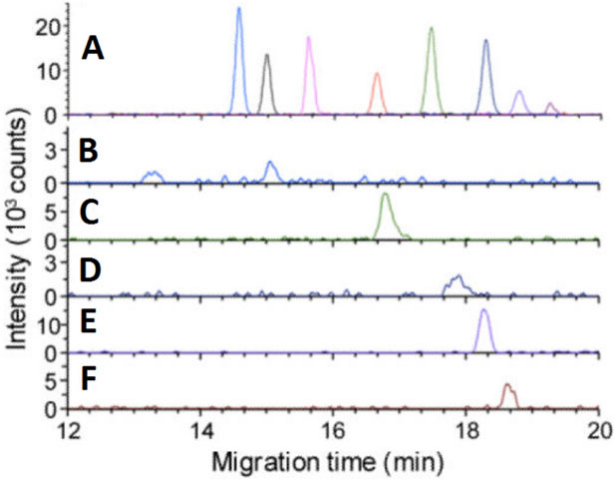
Extracted ion electropherograms obtained with CE–MS using a coaxial sheath‐liquid nanospray interface and a large volume sample injection for (A) nucleotide standards (500 ng/L) and (B) AMP (*m/z* 346.056), (C) ADP (*m/z* 426.022), (D) GDP (*m/z* 442.017), (E) ATP (*m/z* 505.989), and (F) GTP (*m/z* 521.983) nucleotides detected in an extract of an individual neuron from the sea slug *Aplysia californica*. Experimental conditions: BGE, 20 mM ammonium bicarbonate (pH 10); injection volume, 100 nL; separation voltage, +10 kV. Reproduced from 29 with permission.

Liu et al. developed a CE–MS method for the profiling of nucleotides in extracts of single cells using MS in negative‐ionization mode 29. A modified coaxial sheath‐liquid nanospray interface was used that had a smaller diameter capillary outlet, that is 40 μm instead of 75 μm internal diameter, thereby allowing to use sheath‐liquid flow rates below 1 μL/min. These modifications reduced sample dilution and improved detection limits. The interface used in this study was constructed with a microtee assembly containing a platinum alloy emitter in order to prevent corrosion. Various BGEs and sheath‐liquid compositions were evaluated and most optimal analyte responses were obtained with 20 mM ammonium bicarbonate (pH 10) as BGE employing a mixture of isopropanol and water (1:1, v/v) containing 0.2 mM ammonium bicarbonate as sheath‐liquid delivered at a flow rate of 600 nL/min. The method provided a good separation for 16 mono‐, di‐, and triphosphate nucleosides with detection limits ranging from 2 to 22 nM using an injection volume of only 10 nL. To further improve the detection sensitivity of the CE–MS method for the analysis of nucleotides in extracts of an individual neuron from the sea slug *Aplysia californica* a large volume sample stacking procedure was used allowing to inject circa 100 nL (i.e., 14% of the capillary volume). Figure 2 shows that the increased injection volume allowed the detection of five endogenous metabolites (adenosine monophosphate (AMP), ADP, GDP, ATP, and GTP) in a single‐cell extract by the CE–nanospray–MS system, demonstrating the value of this approach for metabolic profiling of ultrasmall biological samples.

**Figure 3:**
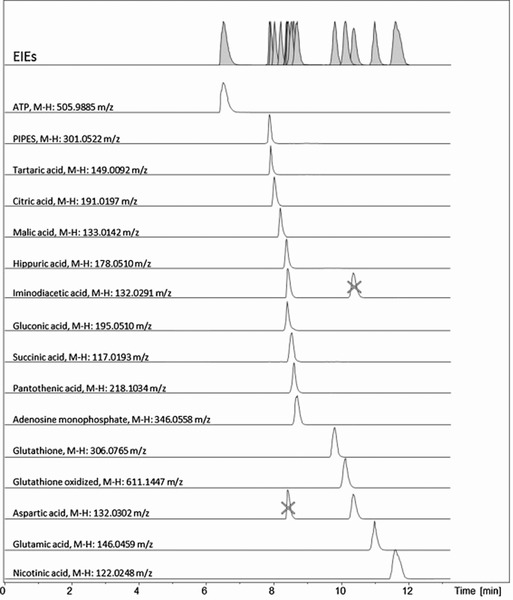
Extracted ion electropherograms obtained with sheath‐liquid CE–MS using a cationic capillary coating for an anionic metabolit mixture containing ATP (0.3 mM), nicotinic acid (7.8 mM), glutamic acid (0.5 mM), aspartic acid (1.0 mM), glutathione oxidized (0.3 mM), glutathione reduced (0.6 mM), iminodiacetic acid (2.7 mM), AMP (0.2 mM), panthotenic acid (0.2 mM), succinic acid (1.4 mM), gluconic acid (0.1 mM), hippuric acid (0.4 mM), malic acid (0.3 mM), citric acid (0.2 mM), tartaric acid (0.2 mM), and 1,4 piperazinediethanesulfonic acid (2.8 mM). Experimental conditions: CE analysis at reverse polarity (−20 kV); BGE, 1 M formic acid (pH 2.4); sample injection, 35 mbar for 80 sec. Reproduced from 33 with permission.

Acunha et al. evaluated poly‐(*N*,*N*,*N*’,*N*’‐tetraethyldiethylenetriamine, *N*‐(2‐hydroxypropyl) methacrylamide (PTH) as a cationic capillary coating for the profiling of anionic metabolites by sheath‐liquid CE–MS 33. The PTH coating provided a strong anodic EOF at low‐pH separation conditions and, therefore, the performance of the method using anionic metabolite mixtures was assessed with acidic BGEs. Anionic metabolites were analyzed by CE in reversed polarity mode and with MS in negative‐ion mode. The use of the PTH coating with 1 M formic acid as BGE allowed the analysis of anionic metabolite standards within 12 min (Fig. 3), whereas with a bare fused‐silica the analysis time was significantly longer and compounds, such as ATP were not observed. Relatively low plate numbers were obtained that may be attributed to the interaction of anionic metabolites with the cationic polymer coating. Potential corrosion with the stainless steel ESI spray needle under these conditions was not reported. Overall, the optimized method allowed the detection of 87 metabolites in orange juice and 142 metabolites in red wine, demonstrating the utility of this approach for the characterization of food products.

The use of coupled SPE CE–MS systems is another way to improve the concentration sensitivity of CE–MS. So far, various groups have shown the utility of either in‐line, that is SPE is an integrated part of CE, or on‐line, that is SPE is coupled to CE via an interface, SPE CE–MS systems for significantly improving the detection limits for compounds such as opioid peptides or drugs in biological samples 34, 35. When combined with a sheathless interface low picomolar detection limits can be obtained 36. Recently, Pont et al. demonstrated the utility of SPE coupled in‐line to CE–MS for the profiling of cationic metabolites in deproteinized plasma samples from mice 37. For in‐line SPE–CE–MS, a home‐made microcartridge containing the SPE C_18_ sorbent was constructed near the inlet of the separation capillary. The performance of the method for cationic metabolic profiling was evaluated with a set of peptides. For metabolic profiling of mice plasma, circa 60 μL of a deproteinized sample was loaded and retained compounds were eluted by injecting a 50 nL solution of methanol/water (6:4, v/v) containing 50 mM acetic acid and 50 mM formic acid. The method was applied to metabolic profiling of plasma from wild‐type mice and of Huntington's disease (HD) mice. The study revealed 29 compounds that were relevant to discriminate between wild‐type and HD plasma samples, as well as to follow‐up HD progression. A single SPE microcartridge could be only used for up to ten analyses of pretreated mice plasma samples. It would be of interest to explore the possibilities of mixed‐mode sorbents for metabolic profiling by in‐line SPE CE–MS as compounds retained on these types of cartridges are better suited for untargeted analysis by CE–MS, whereas with C_18_ sorbents polar and charged metabolites are in part difficult to retain.

## 3 Applications

The applicability of CE–MS for metabolomics in various fields was demonstrated in 32 publications in the period from July 2014 to June 2016. An overview of these studies is given in Table 1, which provides information about the type of sample and compounds analyzed, the BGE, sample pretreatment procedure, the MS analyzer employed, LOD (when provided by the authors), and the type of capillary coating used. In the following sections, representative examples of CE–MS‐based metabolomics are discussed.

**Table 1:** Overview of CE–MS‐based metabolomics studies reported between July 2014 and June 2016

Compounds	Sample matrix	BGE	Sample pretreatment	MS analyzer	LOD	Remarks	Ref.
Non‐targeted applications							
Anionic metabolites	Rat urine	25 mM triethylamine	Dilution with BGE (1:1, v/v)	TOF	0.7–9.1 μM		4
Cationic metabolites	Human breast milk	3 M formic acid	Folch extraction; dried extract dissolved in 20 mM formic acid, followed by centrifugation.	TOF	n.s.		5
Anionic metabolites	Glioblastoma cells	10% acetic acid (pH 2.2)	Methanol/water/chloroform extraction; methanol/water layer evaporated; dried extract reconstituted in water	TOF	10–200 nM	Low‐pH BGE for anionic metabolic profiling; porous tip sheathless interface	32
Cationic metabolites	Orange juice and red wine	1 M formic acid (pH 2.4)	Filtration using 0.2 μm polyethersulfone filter	TOF	0.1–16.4 ppm	Cationic polymer coating for anionic metabolic profiling	33
Cationic metabolites	Mouse plasma	50 mM acetic acid and 50 mM formic acid (pH 3.5)	Cold acetonitrile for protein precipitation followed by centrifugation	TOF	n.s.	C_18_ sorbent for in‐line SPE‐CE	37
Cationic metabolites	Colon cancer cells	3 M formic acid	Water for extraction of intracellular metabolites; cell debris separated by centrifugation	TOF	n.s.		38
Anionic and cationic metabolites	Human serum	50 mM ammonium acetate (pH 8.5); 1 M formic acid (pH 1.8)	Methanol/water/chloroform extraction; methanol/water layer evaporated; dried extract reconstituted in water	TOF	n.s.	Normal CE separation polarity for anionic metabolic profiling; internal standards for quantification	39
Cationic metabolites	Human urine	0.1 M formic acid	Centrifugation and fivefold dilution with separation buffer	TOF	n.s.		40
Cationic metabolites	Mouse plasma	10% acetic acid (pH 2.2)	Cold ethanol for protein precipitation; supernatant evaporated; dried extract reconstituted in 100 mM ammonium acetate (pH 4.0)	TOF	low nM‐range	Neutral coated capillary for separation; porous tip sheathless interface	41
Cationic metabolites	Human plasma	1 M formic acid (pH 1.8) containing 15% acetonitrile	Plasma diluted fourfold with ammonium acetate (pH 5.0); proteins removed using 3‐kDa filter	TOF	n.s.	Multi‐segment injection; internal standard for quantification	43, 56
Anionic and cationic metabolites	Tobacco leaves	50 mM ammonium acetate (pH 8.5); 1 M formic acid (pH 1.8)	Methanol/water/chloroform extraction; methanol/water phase filtered with 5‐kDa ultrafiltration membrane followed by evaporation and reconstitution in water	TOF	n.s.	Normal CE separation polarity for anionic metabolic profiling; internal standards for quantification	44, 45
Anionic and cationic metabolites	*Saccharomyces cerevisiae*	50 mM ammonium acetate (pH 8.5); 1 M formic acid (pH 1.8)	Methanol/water/chloroform extraction; methanol/water layer evaporated; dried extract reconstituted in water	TOF	n.s.	Normal CE separation polarity for anionic metabolic profiling	46
Cationic metabolites	*Leishmania (Leishmania) amazonesis*	0.8 M formic acid containing 10% methanol	Cold methanol/water extraction; supernatant evaporated and reconstituted in water	TOF	n.s.		47
Anionic and cationic metabolites	Porcine muscle	50 mM ammonium acetate (pH 8.5); 1 M formic acid (pH 1.8)	Methanol/water/chloroform extraction; methanol/water layer evaporated; dried extract reconstituted in water	TOF	n.s.	Normal CE separation polarity for anionic metabolic profiling	48
Anionic and cationic metabolites	Colorectal cancer tissues	50 mM ammonium acetate (pH 8.5); 1 M formic acid (pH 1.8)	Methanol/water/chloroform extraction; methanol/water layer evaporated; dried extract reconstituted in water	TOF	n.s.	Normal CE separation polarity for anionic metabolic profiling	49
Anionic and cationic metabolites	Mouse serum and placenta	50 mM ammonium acetate (pH 8.5); 1 M formic acid (pH 1.8)	Methanol/water/chloroform extraction; methanol/water layer evaporated; dried extract reconstituted in water	TOF	n.s.	Normal CE separation polarity for anionic metabolic profiling	50
Anionic and cationic metabolites	Rat and human serum	50 mM ammonium acetate (pH 8.5); 1 M formic acid (pH 1.8)	Methanol/water/chloroform extraction; methanol/water layer evaporated; dried extract reconstituted in water	TOF	n.s.	Normal CE separation polarity for anionic metabolic profiling	51
Anionic and cationic metabolites	Tumor tissues	50 mM ammonium acetate (pH 8.5); 1 M formic acid (pH 1.8)	Methanol/water/chloroform extraction; methanol/water layer evaporated; dried extract reconstituted in water	TOF	n.s.	Normal CE separation polarity for anionic metabolic profiling	52
Anionic and cationic metabolites	Human plasma	50 mM ammonium acetate (pH 8.5); 1 M formic acid (pH 1.8)	Methanol/water/chloroform extraction; methanol/water layer evaporated; dried extract reconstituted in water	TOF	n.s.	Normal CE separation polarity for anionic metabolic profiling	10
Anionic and cationic metabolites	Skeletal muscle	50 mM ammonium acetate (pH 8.5); 1 M formic acid (pH 1.8)	Methanol/water/chloroform extraction; methanol/water layer evaporated; dried extract reconstituted in water	TOF	n.s.	Normal CE separation polarity for anionic metabolic profiling	53
Anionic and cationic metabolites	Rat stomach and serum	50 mM ammonium acetate (pH 8.5); 1 M formic acid (pH 1.8)	Methanol/water/chloroform extraction; methanol/water layer evaporated; dried extract reconstituted in water	TOF	n.s.	Normal CE separation polarity for anionic metabolic profiling	54
Anionic and cationic metabolites	Mouse plasma	50 mM ammonium acetate (pH 8.5); 1 M formic acid (pH 1.8)	Methanol/water/chloroform extraction; methanol/water layer evaporated; dried extract reconstituted in water	TOF	n.s.	Normal CE separation polarity for anionic metabolic profiling	55
Anionic and cationic metabolites	Human plasma and serum	50 mM ammonium acetate (pH 8.5); 1 M formic acid (pH 1.8)	Methanol/water/chloroform extraction; methanol/water layer evaporated; dried extract reconstituted in water	TOF	n.s.	Normal CE separation polarity for anionic metabolic profiling	57
Anionic and cationic metabolites	Fetal hepatocytes	50 mM ammonium acetate (pH 8.5); 1 M formic acid (pH 1.8)	Methanol/water/chloroform extraction; methanol/water layer evaporated; dried extract reconstituted in water	TOF	n.s.	Normal CE separation polarity for anionic metabolic profiling	58
Cationic metabolites	Human serum	0.8 M formic acid containing 10% methanol	Formic acid/acetonitrile extraction; supernatant ultrafiltrated with 30 kDa‐filter	TOF	n.s.		59
Anionic and cationic metabolites	Mouse astrocytes	50 mM ammonium acetate (pH 8.5); 1 M formic acid (pH 1.8)	Methanol/water/chloroform extraction; methanol/water layer evaporated; dried extract reconstituted in water	TOF	n.s.	Normal CE separation polarity for anionic metabolic profiling	60
Cationic metabolites	Embryonic cells	1% M formic acid	Methanol/water containing 0.5% acetic acid for extraction	TOF	low nM‐range	Low‐flow sheath‐liquid interface	61, 62
Targeted							
Nucleotides	*Aplysia californica*	20 mM ammonium bicarbonate (pH 10)	Cold methanol/ammonium bicarbonate (1:1) extraction	TOF	2–22 nM	Low flow sheath‐liquid interface	29
Methylcytosine and hydroxyl‐methylcytosine	Genomic DNA	10% acetic acid (pH 2.2)	Genomic DNA digested; Ultrafiltration with 10‐kDa filter	Triple quadrupole	50–100 pM	Porous tip sheathless interface	42

LOD, S/N = 3; ns, not specified in paper.

### 3.1 Biomedical and clinical applications

Ibánez et al. developed a sheath‐liquid CE–MS method for the profiling of cationic metabolites in extracts of colon cancer HT‐29 cells using a bare fused‐silica capillary and 3 M formic acid as BGE 38. Special attention was devoted to metabolites related to the polyamines pathway due to their role in cell proliferation and carcinogenesis. Four different solvents were evaluated for the extraction of intracellular metabolites from this adherent HT‐29 mammalian cell line, that is, water, water‐formic acid (95:5, v/v), acetonitrile, and isopropyl alcohol‐acetonitrile‐water (3:3:1, v/v/v). Extraction with water provided the highest metabolic coverage with RSDs (*n* = 5) for peak areas of selected metabolites below 9% . The method was used to study metabolic changes in the polyamines pathway produced in colon cancer HT‐29 cells by difluoromethylornithine (DFMO), which is a potent ornithine decarboxylase inhibitor. Ten metabolites including putrescine, ornithine, gamma‐aminobutyric acid, oxidized and reduced glutathione, 5’‐deoxy‐5’‐(methylthio)adenosine, *N*‐acetylputrescine, cysteinyl‐glycine, spermidine and an unknown compound were found to be significantly altered by DFMO (*p* < 0.05) in HT‐29 cells. Besides the effect of DFMO on polyamine metabolism, minor changes of other metabolic pathways were also observed.

Zeng et al. used sheath‐liquid CE–MS to find novel biomarkers for hepatocellular carcinoma (HCC) in human serum samples 39. Cationic metabolites were analyzed at low‐pH separation conditions, whereas anionic metabolites were analyzed at high pH separation conditions using a bare‐fused silica capillary and normal CE polarity in both cases. A total of 183 human serum samples were used in this study including healthy subjects, patients with cirrhosis, and patients with HCC. The study revealed three metabolites, that is, tryptophan, glutamine, and 2‐hydroxybutyric acid that could be reliably used for distinguishing HCC patients from non‐HCC subjects. The diagnostic potential of these three metabolic markers was compared with serum tumor marker alfa‐fetoprotein, which is commonly used in the clinic to diagnose HCC. The comparison indicated that the three metabolic markers has the potential to effectively detect HCC, already at an early stage, showing the utility of CE–MS‐based metabolomics to provide potential diagnostic markers for complicated diseases.

**Figure 4:**
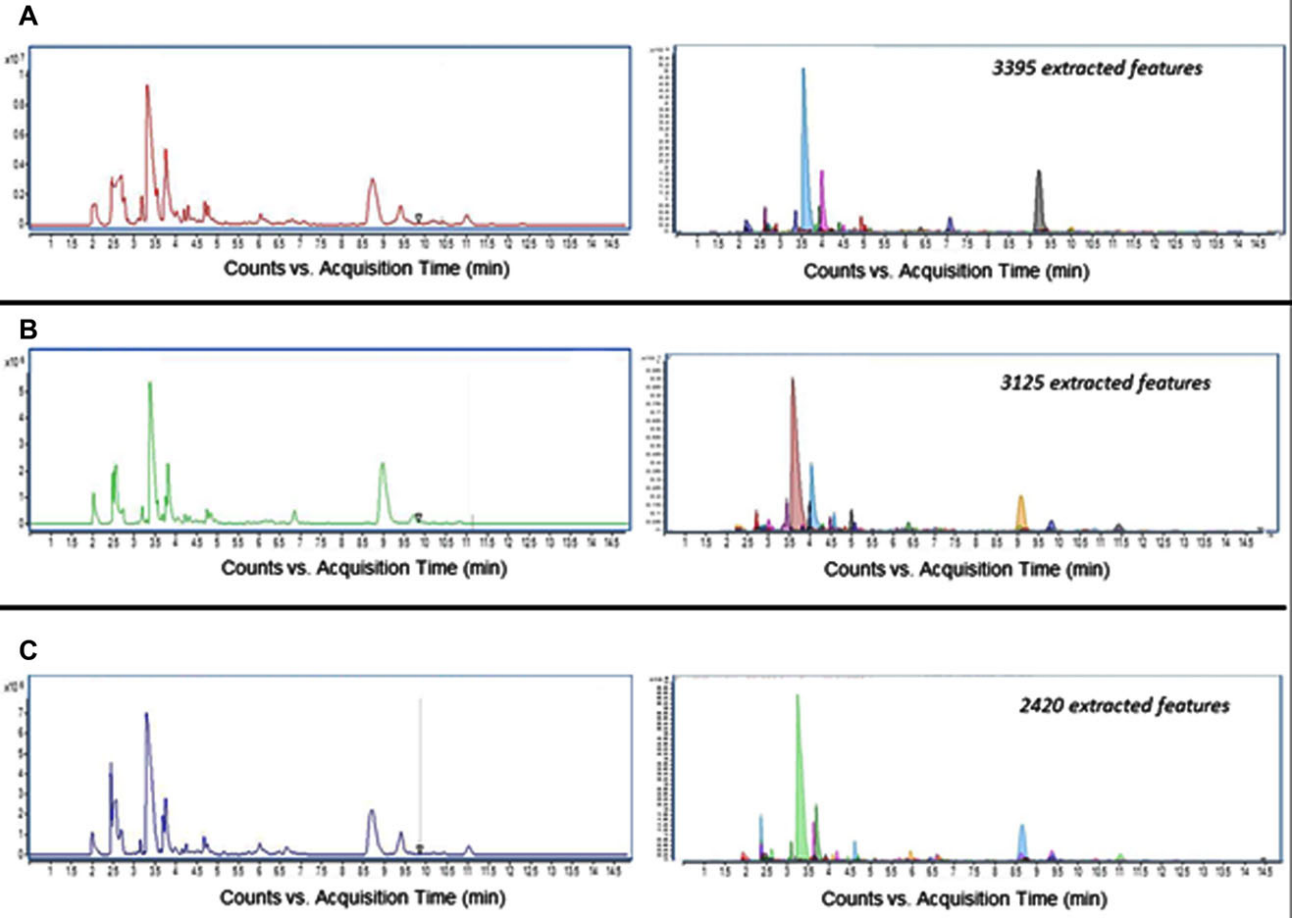
Metabolic profiles obtained by sheath‐liquid CE–MS for three different human urine samples. Shown on the left are total ion electropherograms of (A) a 10 mg tar smoker, (B) a 1 mg tar smoker, and (C) a nonsmoking control subject. On the right are shown the corresponding molecular features extracted from the same 10 mg tar smoker (3395 features), 1 mg tar smoker (3125 features), and nonsmoking (2362 features) urine samples. Experimental conditions: BGE, 0.1 M formic acid; injection volume: 3 nL; separation voltage, +30 kV. Reproduced from 40 with permission.

Garcia‐Perez et al. have used sheath‐liquid CE–MS to study the urinary metabolic profiles of tobacco smokers and nonsmokers 40. The study focused on the global profiling of cationic metabolites at low‐pH separation conditions using a bare fused‐silica capillary. Sample pretreatment of urine comprised centrifugation and a fivefold dilution with BGE (0.1 M formic acid) prior to CE–MS analysis. Typical urinary metabolic profiles obtained by CE–MS for tobacco smokers using two different tar levels and nonsmokers are shown in Fig. 4. Comparison of urinary metabolic profiles of 82 smokers and 39 nonsmokers revealed significant changes in the level of nicotine metabolites. It was also possible to discriminate smokers from nonsmokers based on changes in levels of some endogenous metabolites including glycine and serine, which are intermediates in the metabolism of glutathione. CE–MS covered a set of polar metabolites that were not detected by other analytical platforms, thereby highlighting the potential of this technique for providing potential new biomarkers of tobacco exposure.

**Figure 5:**
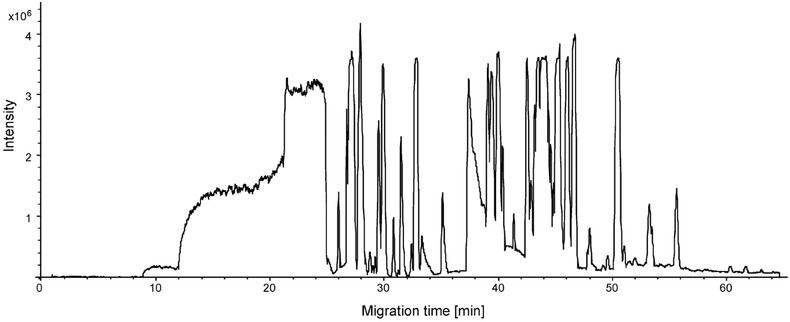
Base peak electropherogram obtained for a deproteinized mouse plasma sample by CE–MS using a sheathless porous tip interface. Experimental conditions: BGE, 10% acetic acid (pH 2.2); sample injection, 25 nL using transient isotachophoresis; separation voltage, +25 kV. Reproduced from 41 with permission.

To allow highly sensitive profiling of cationic metabolites in deproteinized plasma samples from mice, Shyti et al. used a CE–MS method with a sheathless porous tip interface 41. Transient‐isotachophoresis was applied to increase the injection volume and a neutral coated capillary was used for the separation. A typical base peak electropherogram obtained under these conditions for the analysis of a deproteinzed mouse plasma is shown in Fig. 5. The method was used for metabolic profiling of plasma samples obtained from wild‐type mice and transgenic hemiplegic migraine mice after experimentally induced cortical spreading depression. Multivariate data analysis revealed a clear distinction between metabolic profiles of wild‐type and transgenic mice after cortical spreading depression. The amino acid l‐lysine and its by‐product pipecolic acid were found to be mainly responsible for this classification, which was further verified by analysis with hydrophilic interaction chromatography combined with tandem MS. These compounds suggest a compensatory increase in GABAergic neurotransmission upon enhanced excitatory neurotransmission. Overall, this study showed the potential of sheathless CE–MS to provide relevant biochemical information for biological questions dealing with volume‐limited samples.

Yuan et al. developed a CE–MS method using a sheathless porous tip interface for the determination of methylcytosine and hydroxymethylcytosine in genomic DNA 42. The analysis of these compounds is considered challenging due to their low contents within genomic DNA and interferences arising from other nucleosides in this sample. Using an injection volume of 25 nL, sheathless CE–MS provided LODs of 50 and 100 pM for methylcytosine and hydroxymethylcytosine standards, respectively, which was significantly lower as compared to LC–MS methods employing chemical derivatization. The method was used for the determination of these compounds in a genomic DNA extract from mouse embryonic stem cells. Intra‐ and interday RSDs for peak areas were below 6.2 and 7.9%, respectively. Overall, the sheathless CE–MS method was capable of detecting methylcytosine and hydroxymethylcytosine in 125 pg of genomic DNA material dissolved in 16 μL buffer, corresponding to the amount of circa 20 cells. This was significantly less than the amount of genomic DNA sample (10–200 ng) needed for analysis by LC–MS.

Kuehnbaum et al. developed a sheath‐liquid CE–MS method for the profiling of cationic metabolites in human plasma with the aim to find metabolic markers of exercise responsiveness 43. A multisegment injection (MSI) strategy was used for improving sample throughput. MSI–CE–MS using seven serial injections also allowed to assess whether a given molecular feature showed a linear response change (*R* ^2^ > 0.900) upon serial dilution of the biological sample in just a single run. As a result, a list of authentic and reproducible ions could be selected with MSI–CE–MS, thereby acting as a dilution trend filter to exclude redundant ions and background signals. The method was applied to explore adaptive metabolic responses in plasma of overweight/obese women (BMI ˃ 25, *n* = 9) performing a 6‐wk high‐intensity interval training intervention using a repeated measures/cross‐over study design. The study revealed that exercise‐induced increase of l‐carnitine in plasma was indicative of enhanced muscle oxidative capacity, whereas lower circulating plasma thiol redox status reflected greater intracellular antioxidant capacity. Also, adaptive metabolic changes in plasma *O*‐acetyl‐l‐carnitine and hypoxanthine responses postexercise corresponded to lower energetic stress and greater acetylation capacity for trained subjects.

### 3.2 Plant and microbial applications

**Figure 6:**
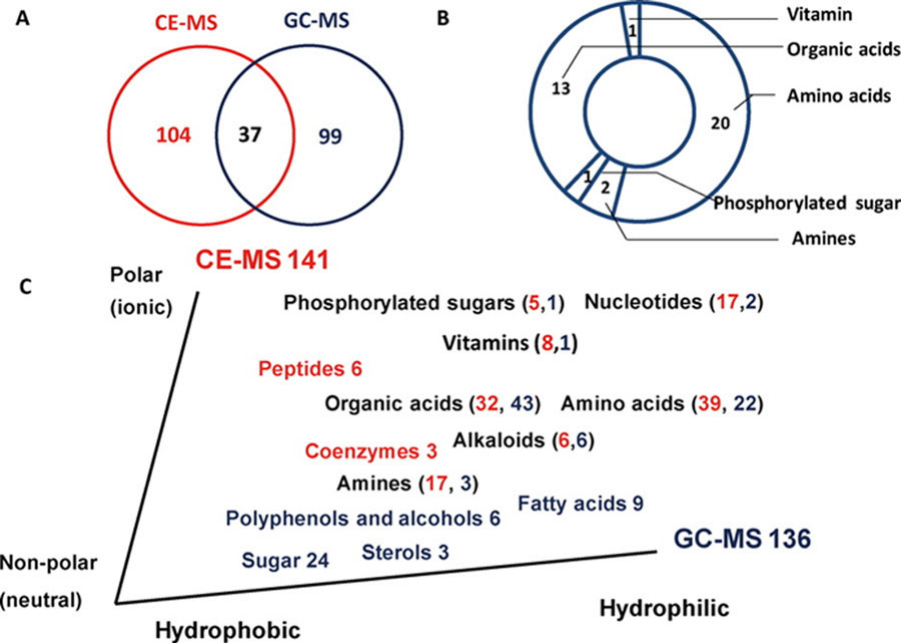
Metabolites identified by CE–MS and GC–MS in a pooled extract of tobacco leaves. (A) Venn diagram of the identified metabolites based on GC–MS and CE–MS. (B) Specific compounds identified by the two platforms. (C) Metabolites that were uniquely detected by CE–MS (red) and GC–MS (blue). Reproduced from 45 with permission.

Ecological conditions may significantly affect the physiological metabolism of plants, still, relatively little is known about the effect of geographical location on dynamic changes in plant leaves during growth. Therefore, Zhao et al. used sheath‐liquid CE–MS and GC–MS to investigate metabolic responses of tobacco leaves to geographical location 44. The use of CE–MS resulted in the identification of 148 metabolites in extracts from tobacco leaves, of which 115 were detected by CE–MS at low‐pH separation conditions and the other 33 metabolites at high pH separation conditions. Of the compounds detected, CE–MS and GC–MS provided an overlap of 37 metabolites, thereby showing the complementarity character of both techniques. For example, GC–MS provided information on sugar and fatty acid metabolism, while CE–MS especially on sugar phosphate, nucleotide, and polyamine metabolism. The study revealed a clear metabolic discrimination between growing districts relative to cultivars. A complex carbon and nitrogen metabolic network was modulated by environmental factors during growth. When Xuchang and Dali Districts in China were compared, results indicated that higher rates of photosynthesis, photorespiration, and respiration were used in the Xuchang district to generate energy and carbon skeletons needed for biosynthesis of nitrogen‐containing metabolites. The same analytical approach was also used to investigate the carbon and nitrogen metabolism of field‐grown tobacco leaves in three major tobacco‐growing provinces in China 45. A total of 277 metabolites were identified in a pooled extract of tobacco leaves, of which 141 by CE–MS and 136 metabolites by GC–MS. Figure 5 shows that only 37 metabolites were detected by both methods, including 20 amino acids and 13 organic acids. Some strongly polar and nonvolatile, metabolites, including 17 nucleotides, 8 vitamins, 5 phosphorylated sugars, 17 amines, 6 peptides, and 3 coenzymes, were only detected by CE–MS, demonstrating the complementary character of CE–MS and GC–MS for metabolic profiling studies (Fig. 6). The study revealed that the growing region greatly influenced the metabolic profiles of tobacco leaves and that carbon‐nitrogen metabolism in tobacco leaves was closely related to regional characteristics.

Nugroho et al. used a CE–MS‐based metabolomics approach in order to understand the effect of lactic acid induced stress on the metabolic composition of *Saccharomyces cerevisiae* 46. For this purpose, cells were cultured with lactic acid, with or without initial pH control, that is, at pH 6 or 2.5, respectively. CE–MS was used for the profiling of intracellular metabolites, employing low‐pH separation conditions for cationic metabolites and high pH separation conditions for anionic metabolites. A bare‐fused silica capillary and normal CE polarity was used under both conditions. The study revealed that the addition of proline to acidified cultures improved specific growth rates. Authors hypothesized that addition of proline protected the cells from acid stress by opposing acid‐induced oxidative stress. Lactic acid diffusion into the cell resulted in intracellular acidification, which elicited an oxidative stress response and resulted in increased glutathione levels.

Castilho‐Martins developed a sheath‐liquid CE–MS method for metabolic profiling of extracts from wild‐type *Leishmania (Leishmania) amazonensis* parasites, abbreviated as *L. (L.) amazonensis*, which were exposed to l‐arginine starvation or not and of extracts from ARG‐knockout mutant *L. (L.) amazonensis*, which lacks the enzyme arginase 47. Cationic metabolites were analyzed with a bare‐fused silica capillary using 0.8 M formic acid containing 10% methanol as BGE. The study revealed that arginine starvation induced a decrease in arginine, ornithine, and putrescine levels, whereas no changes were found for the polyamines spermidine, spermine, and agmatine. However, the absence of the enzyme arginase in ARG‐knockout mutant resulted in an increase of arginine and citrulline levels and in lower levels of ornithine and putrescine. Similarly to the wild‐type arginine‐starved parasites, the ARG‐knockout mutant parasites showed lower levels of proline, thereby possibly indicating an alternative pathway to surpass the enzyme arginase or the lack of substrate.

### 3.3 Food applications

To characterize the metabolic composition of human breast milk, Andreas et al. used a combination of analytical techniques, including RP LC–MS, GC–MS, ^1^H‐NMR, and CE–MS, to profile a wide range of metabolites 5. A total of 710 metabolites spanning multiple molecular classes were defined, of which the majority were identified by RP LC‐‐MS. Amino acids could be selectively analyzed by sheath‐liquid CE‐‐MS at low‐pH separation conditions using a bare fused‐silica capillary. Dynamic changes in breast milk metabolic composition were characterized during the first 3 months of lactation. The multianalytical platform approach revealed that the concentration levels of fucose, di‐, and triacylglycerols, and short‐chain fatty acids significantly changed during the lactation period. These compounds are considered important for infant immunological, neurological, and gastrointestinal development, as well as being an important source of energy.

In order to find metabolites associated with meat quality, Muroya et al. used CE–MS for global profiling of metabolites in postmortem porcine longissimus lumborum (LL) and vastus intermedius (VI) muscles with different aging times 48. Cationic metabolites were analyzed at low‐pH separation conditions, whereas anionic metabolites were analyzed at high pH separation conditions. A bare‐fused silica capillary and normal CE separation polarity was used in both cases. Multivariate data analysis of recorded metabolic profiles revealed that AMP, inosine monophosphate, and inosine changed in a different way during postmortem aging. These results suggest that the presence of enzymes such as adenylate kinase and 5′‐nucleotidase could contribute to the control of pork quality, especially in relation to pork flavor generation.

Details of the remaining CE–MS‐based metabolomics applications of the past two years can be found in Table 1 4, 10, 49, 50, 51, 52, 53, 54, 55, 56, 57, 58, 59, 60, 61, 62.

## 4 Conclusions and perspectives

Over the past 2 years, the applicability of CE–MS for metabolomics studies in various fields was demonstrated in 32 publications, of which a major fraction was focused on the global profiling of metabolites (Table 1). The CE–MS‐based metabolomics studies revealed important findings in the various application areas as outlined in this paper. Though the majority of the reported studies have been performed with CE–MS methods employing a sheath‐liquid interface, there is a growing interest to use the sheathless porous tip interface, especially with respect to further improving the detection sensitivity of CE–MS for metabolomics. The utility of this approach has also been demonstrated for the analysis of anionic metabolites. However, further optimization of this methodology is required in order to allow the highly sensitive profiling of a broad array of anionic metabolites. Furthermore, the analytical performance of sheathless CE–MS for large‐scale clinical studies still need to be determined. Next to the sheathless porous tip interface, various other interfacing designs have been developed over the past few years 63, 64, 65. It would be of great interest to assess their utility for metabolomics studies.

The CE–MS detection sensitivity for metabolic profiling can also be improved by using an in‐line SPE–CE–MS approach. However, further development in this area is needed, especially with respect to the isolation and preconcentration of polar ionogenic metabolites. In this context, it would also be of interest to explore the possibilities of electrodriven sample pretreatment techniques, such as electromembrane extraction and three‐phase electroextraction, for the enrichment of charged compounds 66, 67, 68.

Various distinct advantages of CE–MS for metabolomics studies have been outlined in this paper, for example, a high sample throughput using MSI and the requirement of limited amounts of sample material. It is anticipated that CE–MS will play a key role in those biomedical/clinical studies where sample amounts are severely limited.
